# Correction to: The Fate of Horizontally Acquired Genes: Rapid Initial Turnover Followed by Long-Term Persistence

**DOI:** 10.1093/molbev/msag176

**Published:** 2026-07-24

This is a **correction** to:

Swastik Mishra, Käthe Weit, Martin J Lercher, The Fate of Horizontally Acquired Genes: Rapid Initial Turnover Followed by Long-Term Persistence, *Molecular Biology and Evolution*, Volume 43, Issue 7, July 2026, msag146, https://doi.org/10.1093/molbev/msag146

In the originally published version of this manuscript, there was an error in the funding statement.

This error has been corrected.

